# When Employees Experience Low Levels of Job Autonomy, Fair Procedures Buffer Unfair Outcomes

**DOI:** 10.3389/fpsyg.2022.784853

**Published:** 2022-07-15

**Authors:** Lisanne Versteegt, Marius van Dijke, Joris van Ruysseveldt, Kees van den Bos

**Affiliations:** ^1^Department of Business-Society Management, Rotterdam School of Management, Erasmus University, Rotterdam, Netherlands; ^2^Department of Human Resource Management, Nottingham Business School, Nottingham Trent University, Nottingham, United Kingdom; ^3^Department of Work and Organisational Psychology, Open University of the Netherlands, Heerlen, Netherlands; ^4^Department of Psychology and School of Law, Utrecht University, Utrecht, Netherlands

**Keywords:** distributive justice, procedural justice, job autonomy, job satisfaction, emotional exhaustion

## Abstract

Organizations play a key role in maintaining employee wellbeing. Some research suggests that one way to protect employee wellbeing is to treat them fairly (procedural justice), especially when fair job outcomes (distributive justice) cannot be ensured. Yet, previous studies have not consistently found this interaction effect between distributive and procedural justice. This study investigates job autonomy as a boundary condition to the Distributive Justice × Procedural Justice effect on wellbeing outcomes. To test our hypothesized three-way interaction between distributive justice, procedural justice, and job autonomy, we collected cross-sectional data among Dutch employees in two studies. We used validated self-report measures of our core constructs to test our hypothesis on two employee wellbeing indicators: job satisfaction and emotional exhaustion. Results show a significant three-way interaction effect on both job satisfaction and emotional exhaustion in Study 1 (*N* = 411), and a significant three-way interaction effect on emotional exhaustion in Study 2 (*N* = 1117). Simple slopes analyses of the significant three-way interactions showed that distributive justice and procedural justice interact to predict wellbeing outcomes among employees with low job autonomy. Among employees with high job autonomy, distributive justice and procedural justice do not interact to predict wellbeing. The results contribute to the employee wellbeing literature by showing that job autonomy is a boundary condition to the Distributive Justice × Procedural Justice effect on wellbeing outcomes. We discuss other implications of our findings for the workplace and the ramifications for employees with low and high job autonomy.

## Introduction

Organizations play a significant role in maintaining the wellbeing of their employees. One of the most predominant ways in which organizations affect wellbeing is through organizational justice (see, e.g., Cohen-Charash and Spector, 2001; Colquitt et al., 2001, 2013; Robbins et al., 2012). In particular, two antecedents of justice in organizations are often distinguished: distributive justice (the fairness of outcomes as perceived by employees) and procedural justice (the fairness of procedures as perceived by employees). Research shows that these types of justice judgments affect wellbeing outcomes; for instance, perceiving to be underpaid (i.e., low distributive justice) increases levels of stress (Greenberg, 2006), while having an opportunity to voice opinions about decision-making processes (i.e., high procedural justice) decreases levels of stress (Brotheridge, 2003).

While there is continuing interest in the relationship between distributive justice, procedural justice, and employee wellbeing (Sheeraz et al., 2021), recent work has mostly focused on the main effects of these forms of justice on wellbeing outcomes (e.g., Bakotić and Bulog, 2021). Yet, one intriguing finding in the organizational justice literature is that distributive and procedural justice interact to influence a broad range of organizational outcomes (for overviews, see Brockner and Wiesenfeld, 1996, 2005; Brockner, 2011). This interaction is often described as a buffering interaction effect; that is, high procedural justice buffers the negative effects of low distributive justice. This interaction effect indicates that organizations can reduce the negative effects of unfair outcomes on wellbeing by increasing procedural justice; for instance, to minimize stress, organizations could provide voice to employees when outcomes are unfair (Vermunt and Steensma, 2003; Brockner, 2011). As organizations have significant control over the fairness of procedures, understanding the nature of this interaction effect on employee wellbeing outcomes could increase the organization's ability to support employees' welfare.

Despite the important conceptual and practical contribution of the Distributive Justice × Procedural Justice interaction to the employee wellbeing literature (Brockner, 2011), few studies have tested this interaction effect on wellbeing outcomes (for reviews, see Vermunt and Steensma, 2005; Brockner, 2011). Furthermore, in those few studies that did test the interaction effect, the interaction has failed to consistently materialize: While some papers found a significant interaction on wellbeing outcomes such as job satisfaction (Fields et al., 2000) and emotional exhaustion (Tepper, 2001), other studies did not find this interaction effect on similar outcomes (McFarlin and Sweeney, 1992; Fischer et al., 2014). One explanation for this inconsistency is that there are boundary conditions to the effect that are currently not fully understood. Identifying boundary conditions of the effect is important because it helps explain *why* fair procedures buffer against the negative consequences of unfair outcomes. From a practical perspective, because employees often experience injustice, knowing *when* the interaction materializes will help organizations safeguard employees' wellbeing at work by understanding better when it is especially important to ensure that processes are fair.

In this paper, we propose that the extent to which employees can make autonomous decisions in their job is a boundary condition to the Distributive Justice × Procedural Justice interaction on wellbeing. We focus on job autonomy. This concept (often also referred to as job control; Karasek, 1979) is a core construct in job wellbeing literature with well-established benefits for wellbeing outcomes (e.g., Humphrey et al., 2007). Previous research showed that job autonomy attenuates the negative consequences of distributive injustice (Haar and Spell, 2009) and procedural injustice (Rousseau et al., 2009), suggesting that having high levels of job autonomy protects employees' wellbeing when they are confronted with unfair outcomes or unfair procedures. Yet, some jobs are characterized by low levels of autonomy—examples include machine operators, assemblers, clerks, and cashiers (Vidal, 2013). Additionally, low autonomous jobs also tend to be low-wage jobs (Vidal, 2013), making it more likely for employees in these jobs to experience low distributive justice. While organizational justice research suggests that procedural justice can buffer the negative consequences of distributive injustice, it is unclear to what extent employees with low levels of job autonomy may benefit from fair procedures. We will argue that the Distributive Justice × Procedural Justice interaction is more likely to emerge for employees who have low, rather than high job autonomy. In developing our theoretical reasoning, we take inspiration from the literature on wellbeing.

## Theoretical Background and Hypothesis Development

### The Distributive × Procedural Justice Interaction and Employee Wellbeing

Wellbeing typically refers to an employee's work satisfaction and state of mental and physical health (Danna and Griffin, 1999). Research on the job demand-control (JDC) model (Karasek, 1979) shows that employee wellbeing is affected by the joint effects of two work elements: job demands and job control (Karasek, 1979; Theorell and Karasek, 1996). Job demands refer to stressors in the job and work environment, such as a high workload or workplace conflicts, that require employees to exert effort and engage in action to respond to these demands (Karasek, 1979). When employees experience high job demands, they are more likely to experience stress and strain, which negatively impacts their wellbeing (Gonzalez-Mulé et al., 2021). Job autonomy or job control (e.g., Karasek, 1979) refers to the extent to which employees have freedom, independence, and personal discretion to carry out their work (Breaugh, 1985).

The central tenet of the JDC model is that the combination of job demands and job control predicts wellbeing outcomes. One important joint effect is the buffer effect (Gonzalez-Mulé et al., 2021); job control buffers against the negative effects of job demands on wellbeing. Poor wellbeing is more likely when employees experience high demands in combination with low job control. That is, having low control decreases an employee's ability to cope with job demands (Karasek, 1979). However, when employees have more control over the way they respond to stressors they are, overall, better equipped to resolve job demands.

We note here that the literature on organizational justice proposes a similar process with respect to how people respond to experiences of distributive and procedural justice (Brockner, 2011). Specifically, similar to how appropriate levels of job control buffer against high job demands or other stressors (Karasek, 1979), so can procedural justice buffer against the stressor of distributive injustice. As explained by equity theory (Adams, 1965), unfair outcomes constitute a direct personal loss to the person who is disadvantaged and creates tension and dissatisfaction. This claim is supported by research showing that unfair outcomes invoke discrete negative emotions, such as anger (Weiss et al., 1999), and also, more broadly, negative affect (Colquitt et al., 2013). Distributive injustice can therefore be viewed as a stressor that may lead to poor wellbeing, especially when control or autonomy is low (for a meta-analysis, see Robbins et al., 2012).

One way in which procedural justice may buffer against the stressor of distributive injustice is by supporting a feeling of control (Thibaut and Walker, 1975; Judge and Colquitt, 2004). In their instrumental model of procedural justice, Thibaut and Walker (1975) argued that process control is key to procedural justice. That is, people desire to have control over the processes by which decisions are made, and processes are seen as more fair when some control can be exercised. This instrumental model of procedural justice, therefore, suggests that when processes are fair, employees are less affected by the stressor of unfair outcomes as they perceive some control over future outcomes.

A few studies show that distributive justice and procedural justice interact to affect or predict different indicators of employee wellbeing in the direction that is hypothesized by the JDC model (for a review, see Brockner, 2011). For instance, Tepper (2001) found that employees experiencing unfair outcomes reported the highest levels of depression, anxiety, and emotional exhaustion when procedures were also unfair. In other words, experiencing a job stressor (such as low levels of distributive justice) combined with perceiving little control (such as is the case when people experience low levels of procedural justice) is likely to lead to the lowest levels of employee wellbeing. In contrast, perceiving some control (such as higher levels of procedural justice) may well buffer against the stressor of unfair outcomes. Fields et al. (2000) indeed showed that the combination of low procedural and distributive justice predicted the lowest levels of job satisfaction, while high procedural justice weakened the negative effect of low distributive justice on job satisfaction. In the present paper we build on these insights to propose that the Distributive Justice × Procedural Justice interaction on wellbeing-related outcomes is more pronounced among employees with low (vs. high) job autonomy.

### The Role of Job Autonomy

Following the logic of the buffering effect in the JDC model (Karasek, 1979; Gonzalez-Mulé et al., 2021), we argue that job autonomy mitigates the effects of stressors (i.e., demands), such as distributive injustice, and compensates for low process control, such as resulting from procedural injustice. Previous research showing that high (vs. low) job autonomy protects against the effect of low (vs. high) distributive justice (Haar and Spell, 2009), and low (vs. high) procedural justice (Rousseau et al., 2009), supports this expectation.

We extend this prior research that has looked at the role of job autonomy in effects of distributive or procedural justice in isolation by arguing that low job autonomy increases the importance of procedural justice when distributive justice is low. Employees with low job autonomy have less control over the way they respond to stressors encountered at work (Bakker et al., 2005), such as unfair outcomes. As procedural justice makes outcomes feel more controllable and predictable (Thibaut and Walker, 1975), employees with low job autonomy (i.e., with low job control) may rely more on procedural justice to cope with low distributive justice. Work by Van Prooijen (2009) supports this expectation by showing that people with low levels of autonomy rely more on procedural justice judgments than those with high levels of autonomy. In other words, when employees with low job autonomy are confronted with unfair outcomes, high procedural justice leads them to perceive some control that helps them cope with the stressor of such outcomes. Employees with high job autonomy need to rely less on procedural justice when they are confronted with unfair outcomes because job autonomy provides them more control over the way that they respond to and cope with these demands (Karasek, 1979; Bakker et al., 2005). Thus, we expect that the Distributive Justice × Procedural Justice interaction emerges for employees with low levels of job autonomy and not for employees with high levels of job autonomy.

As indirect support that job autonomy may moderate the Distributive Justice × Procedural Justice interaction, previous work shows that this interaction effect is especially pronounced among organization members in lower power and status positions (Chen et al., 2003; Blader and Chen, 2012; Bianchi et al., 2015; Van Dijke et al., 2019), who may also experience lower autonomy. Yet, job autonomy and power are distinct constructs. Job autonomy refers to the extent to which employees have control over their work and therefore the ability to respond to job demands (Karasek, 1979), while power refers to controlling the outcomes of others (Lammers et al., 2016). Furthermore, the effect of power is often explained in terms of trust: those in lower power positions rely on justice to judge whether those in higher power positions can be trusted to not abuse their power (e.g., Bianchi et al., 2015; Van Dijke et al., 2019). In contrast, the current study focuses on employees' control over how and when to respond to the demands of injustice. Taken together, our reasoning leads to the following hypothesis:

*Employee's job autonomy moderates the interaction effect of distributive and procedural justice on job wellbeing such that the Distributive Justice* × *Procedural Justice interaction will be stronger when employee's job autonomy is low (vs. high)*.

## Study Overview

We tested the hypothesized three-way interaction of distributive justice, procedural justice, and job autonomy on two different but related indicators of employee wellbeing: job satisfaction and emotional exhaustion. Job satisfaction is a common operationalization of work-related wellbeing (Danna and Griffin, 1999), and a commonly used outcome in organizational justice research. Emotional exhaustion, a key component of burnout (Maslach and Jackson, 1981), is characterized by feeling emotionally drained and mentally fatigued. It is therefore considered an important factor in employees' wellbeing. Previous work has suggested that emotional exhaustion is a result of having insufficient control to cope with job demands or job stressors (e.g., Bakker et al., 2005). Against this background, we conducted two field studies. In Study 1, we asked Dutch employees to respond to a questionnaire with validated measures of our key constructs. In Study 2, we examined the robustness of our findings by increasing the sample size and using more extensive measures.

### Study 1

#### Method

##### Respondents and Procedure

We recruited employees from a variety of organizations in the Netherlands *via* Flycatcher, a Dutch research panel consisting of over 10,000 Dutch citizens. Flycatcher complies with strict quality requirements for research and has ISO-certification (i.e., it meets the qualitative ISO requirements for social scientific research). Members of Flycatcher who worked for at least 12 h each week were invited to fill out a questionnaire on a web page. For their participation, respondents received credit points that would allow them to receive certain gifts (e.g., tickets for the movies).

We aimed to obtain a sample that was as representative of Dutch employees as possible while working with a panel. To obtain such a sample, Flycatcher used a stratified sampling approach based on data provided by the Central Office for Statistics of the Netherlands regarding gender, age, and education. Of the invited (*N* = 422), 97.6% (*N* = 412) completed the questionnaire. The sample of Study 1 is described in Table 1 together with data from the Central Office for Statistics (Centraal Bureau Statistiek, 2022). Chi-squared tests showed that the expected proportions based on COS data were significantly different from our sample. The main differences are found in men being somewhat overrepresented in our data, younger (15–19 and 20–24 years old) and older (60–64 years old) workers being somewhat underrepresented, and higher educated workers being overrepresented while lower educated workers were underrepresented. We therefore control for these variables in our robustness check analyses in the results section.

**Table 1:** Sample description Study 1.

		**Study 1**		**COS**
**Variable**	**Category**	***N***	***%***	***%***
Gender	Male	259	62.9	49.5
	Female	153	37.1	50.5
Highest completed level of education	Lower education (high school degree and lower)	82	19.9	32.9
	Vocational education	182	44.2	38.9
	Higher education (bachelor's degree and higher)	148	35.9	27.3
	Unknown	0		0.9
Age	15–19	2	0.5	9.0
	20–24	24	5.8	9.4
	25–29	46	11.2	9.1
	30–34	58	14.1	9.1
	35–39	48	11.7	9.6
	40–44	63	15.3	11.6
	45–49	61	14.8	11.7
	50–54	59	14.3	10.9
	55–59	43	10.4	9.9
	60–64	8	1.9	9.6
Work hours per week	20–35	155	37.6	
	36 or more	257	62.4	

*N = 412. COS, Central Office for Statistics of the Netherlands*.

##### Measures

We assessed *procedural justice* using Colquitt's validated seven-item scale (Colquitt, 2001). Respondents answered on a 5-point Likert-type scale ranging from 1 (*strongly disagree)* to 5 (*strongly agree*). An example item is “The procedures used to determine my salary are based on accurate information.”

We measured *distributive justice* with Colquitt's (2001) four-item distributive justice scale. Respondents answered using a 5-point Likert-type scale ranging from 1 (*to a small extent*) to 5 (*to a large extent*). An example item is “Does your salary reflect the effort you have put into your work?”

We measured *job autonomy* with three items from the decision latitude subscale in Karasek (1985) Job Content Questionnaire [adapted and translated by Goudswaard et al. (1998)]. Respondents answered on a 4-point Likert-type scale ranging from 1 (*never*) to 4 (*always*). One of the items is “Can you decide yourself how to execute your job?”

We assessed *job satisfaction* with a one-item job satisfaction measure. We used a one-item measure to shorten the questionnaire. Research indicates that one-item measures of general job satisfaction are valid and reliable (Wanous et al., 1997; De Jonge and Schaufeli, 1998). This measure asked respondents, “To what extent are you, generally speaking, satisfied with this job?” Responses were given on a 5-point Likert-type scale ranging from 1 (*very dissatisfied*) to 5 (*very satisfied*).

Finally, we measured *emotional exhaustion* with a 5-item subscale of the Dutch version of the Maslach Burnout Inventory (MBI; Maslach and Jackson, 1986), the MBI-NL-ES (Schaufeli et al., 1994; Horn and van Schaufeli, 1998). One of the items is “I feel used up by the end of the day.” Respondents answered on a 7-point Likert-type scale ranging from 1 (*never*) to 7 (*always*).

#### Results

##### Main Analyses

We screened the data for outliers following recommendations from Aguinis et al. (2013). Multivariate outlier analyses revealed one case with a large leverage value (0.16) and studentized residuals (-2.97). Influential outlier tests using Cook's distance (0.21) and difference in fits (DFFITS; −1.22) further indicated that this case was an outlier. Investigation of this case showed that scores were extremely low for all variables (this participant always responded with “1”, which implies 2.19, 2.67, 2.57 and 3.45 *SD* below the mean for distributive justice, procedural justice, job autonomy, and job satisfaction, respectively) except for emotional exhaustion (0.59 *SD* below the mean). We report the results obtained after filtering out this influential outlier. Sensitivity analysis revealed that our final sample of 411 participants allowed us to detect a small effect size (*f*^2^ = 0.02; Cohen, 1988) with 80% power (Faul et al., 2009). Table 2 presents scale means, standard deviations, Cronbach's alpha coefficients, and intercorrelations (*N* = 411). We mean-centered our predictor variables prior to analyses (Aiken and West, 1991).

**Table 2:** Means, standard deviations, intercorrelations, and Cronbach's alpha coefficients of Study 1 variables.

**Variables**	***M***	***SD***	**1**	**2**	**3**	**4**	**5**
1. Distributive justice	2.89	0.85	**0.95**				
2. Procedural justice	2.98	0.73	0.43 (<0.001)	**0.86**			
3. Job autonomy	2.90	0.74	0.08 (0.118)	0.24 (<0.001)	**0.88**		
4. Job satisfaction	3.84	0.81	0.32 (<0.001)	0.37 (<0.001)	0.34 (<0.001)		
5. Emotional exhaustion	2.99	1.34	−0.18 (<0.001)	−0.23 (<0.001)	−0.31 (<0.001)	−0.57 (<0.001)	**0.94**

*N = 411. Numbers in parentheses are corresponding p-values. The numbers in bold are the Cronbach's α*.

We tested our hypotheses using Ordinary Least Squares (OLS) regressions, with job satisfaction and emotional exhaustion as outcome variables. We tested three models. In model 1, we entered the main effects of distributive justice, procedural justice, and job autonomy. In model 2, we entered the first-order interactions between these variables. In model 3, we entered the three-way interaction that was of primary interest in the present research. Table 3 presents the results. We found significant three-way interactions between distributive justice, procedural justice, and job autonomy on job satisfaction and emotional exhaustion.

**Table 3:** OLS regression results of job satisfaction and emotional exhaustion on distributive justice, procedural justice, and job autonomy (Study 1).

	**Job satisfaction**						**Emotional exhaustion**					
	**Model 1**		**Model 2**		**Model 3**		**Model 1**		**Model 2**		**Model 3**	
	***B***	***SE***	***B***	***SE***	***B***	***SE***	***B***	***SE***	***B***	***SE***	***B***	***SE***
Intercept	3.84 (<0.001)	0.03	3.87 (<0.001)	0.04	3.86 (<0.001)	0.04	2.99 (<0.001)	0.06	2.92 (<0.001)	0.07	2.92 (<0.001)	0.07
DJ	0.19 (<0.001)	0.05	0.19 (<0.001)	0.05	0.17 (<0.001)	0.05	−0.18 (0.028)	0.08	−0.18 (0.027)	0.08	−0.15 (0.067)	0.08
PJ	0.25 (<0.001)	0.05	0.27 (<0.001)	0.06	0.26 (<0.001)	0.06	−0.21 (0.029)	0.10	−0.21 (0.030)	0.10	−0.19 (0.048)	0.10
AUT	0.30 (<0.001)	0.05	0.25 (<0.001)	0.05	0.21 (<0.001)	0.05	−0.50 (<0.001)	0.09	−0.45 (<0.001)	0.09	−0.39 (<0.001)	0.09
DJ × PJ			−0.01 (0.790)	0.05	−0.06 (0.242)	0.05			0.14 (0.129)	0.09	0.23 (0.019)	0.10
PJ × AUT			−0.19 (0.004)	0.07	−0.15 (0.029)	0.07			0.24 (0.041)	0.12	0.17 (0.159)	0.12
DJ × AUT			−0.01 (0.927)	0.06	0.05 (0.456)	0.06			−0.00 (0.980)	0.11	−0.10 (0.405)	0.11
DJ × PJ × AUT					0.19 (0.003)	0.06					−0.32 (0.005)	0.11
*R^2^*	0.24 (<0.001)		0.26 (<0.001)		0.27 (<0.001)		0.13 (<0.001)		0.15 (<0.001)		0.17 (<0.001)	
*R^2^ change*			0.02 (0.014)		0.01 (0.003)				0.02 (0.046)		0.02 (0.005)	

*N = 411. All predictors were centered on the grand mean prior to analyses. Numbers in parentheses are corresponding p-values. DJ, distributive justice; PJ, procedural justice; AUT, job autonomy*.

**Figure 1:**
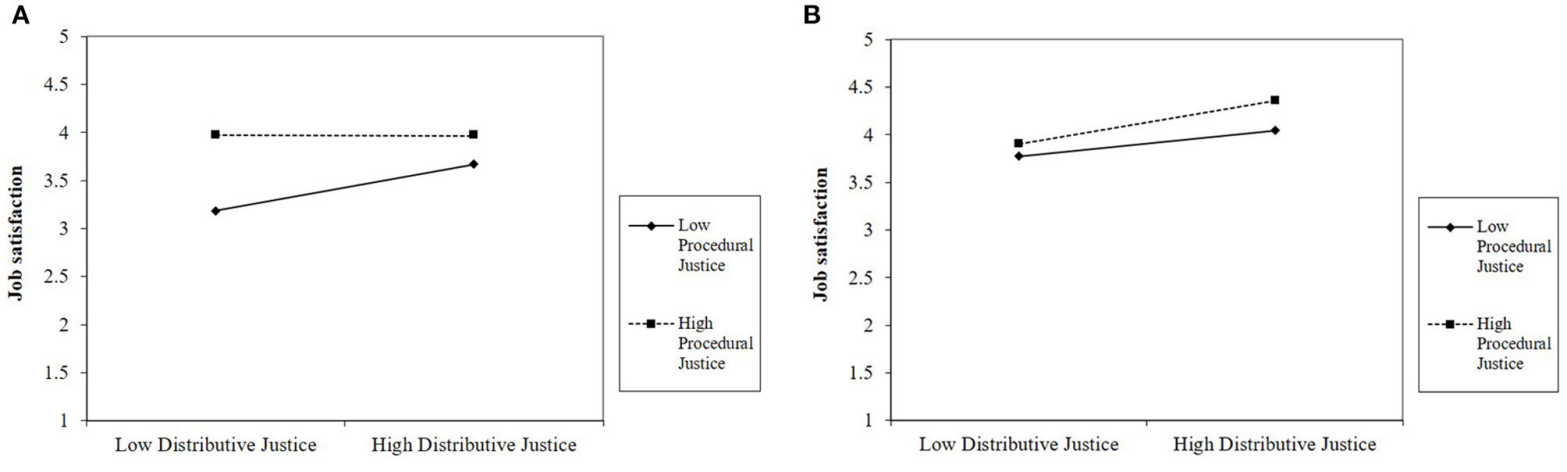
**(A)** The effect of distributive and procedural justice on job satisfaction when job autonomy is low (Study 1). **(B)** The effect of distributive and procedural justice on job satisfaction when job autonomy is high (Study 1).

Figures 1A,B depict the interaction for job satisfaction. Simple slopes analyses using the PROCESS macro (version 3.4) for SPSS (Model 3; Hayes, 2018) showed that among employees with low job autonomy (1 *SD* below the mean on autonomy), the Distributive Justice × Procedural Justice interaction was significant (*B* = −0.20, *F*_(1,403)_ = 6.01, *p* = 0.015). More specifically, among employees with low job autonomy, distributive justice was positively related to job satisfaction when procedural justice was low (1 *SD* below the mean; *B* = 0.28, *SE* = 0.08, *p* < 0.001) but not significantly related when procedural justice was high (1 *SD* above the mean; *B* = −0.01, *SE* = 0.10, *p* = 0.928). From a different vantage point, among employees with low autonomy, the effect of procedural justice was significant when distributive justice was low (*B* = 0.54, *SE* = 0.09, *p* < 0.001) and not significant when distributive justice was high (*B* = 0.20, *SE* = 0.12, *p* = *0.0*99). Among employees with high job autonomy, the Distributive Justice × Procedural justice interaction was not significant (*B* = 0.07, *F*_(1,403)_ = 1.52, *p* = 0.218).

**Figure 2:**
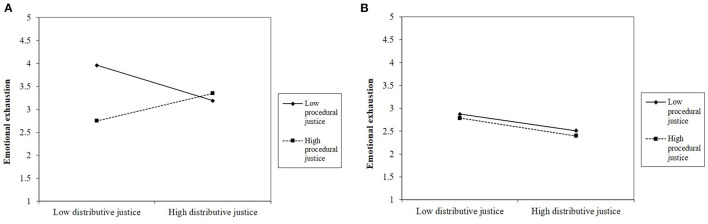
**(A)** The effect of distributive and procedural justice on emotional exhaustion when job autonomy is low (Study 1). **(B)** The effect of distributive and procedural justice on emotional exhaustion when job autonomy is high (Study 1).

Figures 2A,B depict the interaction for emotional exhaustion. Simple slopes analyses showed that among employees with low job autonomy (1 *SD* below the mean), the interaction between distributive and procedural justice was significant (*B* = 0.46, *F*_(1,403)_ = 10.15, *p* = 0.002). More specifically, among employees with low job autonomy, distributive justice was negatively related to emotional exhaustion when procedural justice was low (1 *SD* below the mean; *B* = −0.42, *SE* = 0.14, *p* = 0.003) but not significantly related when procedural justice was high (1 *SD* above the mean; *B* = 0.26, *SE* = 0.18, *p* = 0.156). From a different vantage point, among employees with low autonomy, the effect of procedural justice was significant when distributive justice was low (*B* = −0.71, *SE* = 0.16, *p* < 0.001) and not significant when distributive justice was high (*B* = 0.08, *SE* = 0.21, *p* = *0.7*19). Among employees with high job autonomy, the Distributive Justice × Procedural justice interaction was not significant (*B* = −0.01, *F*_(1,403)_ = 0.01, *p* = 0.927).

##### Robustness Checks

We followed the recommendations from Spector and Brannick (2011) and first estimated models that did not include demographic variables as controls. As a robustness check, we estimated the same three models but included gender, age, and education level as predictors. The focal three-way interaction on job satisfaction (*B* = 0.18, *SE* = 0.08, *p* = 0.004) and emotional exhaustion (*B* = −0.29, *SE* = 0.11, *p* = 0.008) was still significant and of the same shape as in the main analyses section.

We conducted the same OLS regression analyses as in the main analyses section including the participant who responded with “1” on all variables except emotional exhaustion. The results showed a nonsignificant three-way interaction effect for emotional exhaustion in the expected direction (*B* = −0.14, *SE* = 0.10, *p* = 0.165), while the level of significance for the effect of the three-way interaction on job satisfaction remained unchanged (*B* = 0.18, *SE* = 0.06, *p* = 0.002).

### Study 2

Study 1 supports our hypothesis that the Distributive Justice × Procedural justice interaction on the two wellbeing outcomes, emotional exhaustion and job satisfaction, is pronounced most among employees who have low (vs. high) job autonomy.

To test the robustness of our findings, we conducted a second study, in which we introduced some changes. First, some results in Study 1 were affected by one influential outlier. As larger sample sizes reduce the influence of individual points (i.e., outliers; Belsley et al., 1980), we increased the sample size in Study 2 to decrease the influence of individual cases. Second, we wanted to test if the results obtained on the single-item job satisfaction measure in Study 1 can be replicated with a multi-item measure of the same construct, which we included in Study 2. Finally, in Study 2 we also used more extensive measures of job autonomy and emotional exhaustion.

#### Method

##### Respondents and Procedure

As in Study 1, the respondents were recruited from Flycatcher. Of the invited (*N* = 1278), 96.2% (*N* = 1229) completed the questionnaire. We excluded those who had participated in Study 1 (9.1%), resulting in a final sample of *N* = 1117. The sample of Study 2 is described in Table 4 together with data from the Central Office for Statistics (Centraal Bureau Statistiek, 2022). Chi-squared tests showed that the expected proportions based on COS data were not significantly different from our sample for gender and highest completed level of education. For age, the proportions were significantly different. The main differences are found in younger (15–19, 20–24) and older (60–64) workers being underrepresented in our data. We therefore control for these variables in our robustness check analyses in the results section.

**Table 4:** Sample description Study 2.

**Categorical variables**		**Study 2**		**COS**
**Variable**	**Category**	***N***	***%***	***%***
Gender	Male	558	50.0	49.5
	Female	559	50.0	50.5
Highest completed level of education	Lower education (high school degree and lower)	361	32.3	32.9
	Vocational education	435	38.9	38.9
	Higher education (bachelor's degree and higher)	321	28.7	27.3
	Unknown	0	0.0	0.9
Age	15–19	0	0.0	9.0
	20–24	0	0.0	9.4
	25–29	121	10.8	9.1
	30–34	141	12.6	9.1
	35–39	146	13.1	9.6
	40–44	169	15.1	11.6
	45–49	173	15.5	11.7
	50–54	151	13.5	10.9
	55–59	156	14.0	9.9
	60–64	60	5.4	9.6
Continuous variables				
Variable	Range	Average	*SD*	
Work hours per week	0–40	29.92	10.39	

*N = 1117. COS = Central Office for Statistics in the Netherlands*.

#### Measures

We assessed *distributive justice* and *procedural justice* with the same scales as in Study 1.

We measured *job autonomy* with a 5-item scale validated in Dutch, which was adapted from the decision latitude subscale in Karasek's Job Content Questionnaire (Goudswaard et al., 1998). An example item is “Can you decide yourself how to execute a task?” Respondents answered on a 5-point Likert-type scale ranging from 1 (*never*) to 5 (*always*).

We measured *job satisfaction* with a 4-item scale from Brayfield and Rothe (1951), which has been validated in Dutch (Guest et al., 2010). An example item is “Most days I am enthusiastic about my work.” Respondents answered on a 5-point scale ranging from 1 (*totally disagree*) to 5 (*totally agree*).

Finally, we measured *emotional exhaustion* with the 8-item subscale of the Dutch version of the Maslach Burnout Inventory (MBI; Maslach and Jackson, 1986), the MBI-NL-ES (Schaufeli et al., 1994; Horn and van Schaufeli, 1998). An example item is “How often do you feel emotionally drained from your work?” Respondents answered on a 7-point Likert-type scale ranging from 1 (*never*) to 7 (*always*).

#### Results

##### Main Analyses

We screened the data for outliers as we did in Study 1. Multivariate outlier analyses did not reveal any case with relatively large leverage values and studentized residuals nor did tests with Cook's distance and difference in fits (DFFITS) values indicate any outlier. We therefore retained all cases for the analyses. Table 5 presents scale means, standard deviations, Cronbach's alpha coefficients, and intercorrelations. We mean-centered our predictor variables prior to analyses (Aiken and West, 1991).

**Table 5:** Means, standard deviations, intercorrelations, and Cronbach's alpha coefficients of Study 2 variables.

**Variables**	***M***	***SD***	**1**	**2**	**3**	**4**	**5**
1. Distributive justice	2.78	0.89	**0.96**				
2. Procedural justice	2.66	0.83	0.40 (<0.001)	**0.89**			
3. Job autonomy	3.81	0.79	0.07 (0.031)	0.15 (<0.001)	**0.86**		
4. Job satisfaction	3.98	0.84	0.18 (<0.001)	0.27 (<0.001)	0.15 (<0.001)	**0.90**	
5. Emotional exhaustion	2.66	0.99	−0.21 (<0.001)	−0.20 (<0.001)	−0.11 (<0.001)	−0.42 (<0.001)	**0.90**

*N = 1117. Numbers in parentheses are corresponding p-values. The numbers in bold are the Cronbach's α*.

We tested our hypothesis using the same OLS regression procedures as in Study 1. Table 6 presents the results. We found a significant three-way interaction between distributive justice, procedural justice, and job autonomy for emotional exhaustion (*B* = −0.12, *SE* = 0.04, *p* = 0.005). The three-way interaction was in the expected direction, but it did not reach significance for job satisfaction (*B* = 0.04, *SE* = 0.04, *p* = 0.252).

**Table 6:** OLS regression results of job satisfaction and emotional exhaustion on distributive justice, procedural justice, and job autonomy (Study 2).

	**Job satisfaction**						**Emotional exhaustion**					
	**Model 1**		**Model 2**		**Model 3**		**Model 1**		**Model 2**		**Model 3**	
	***B***	***SE***	***B***	***SE***	***B***	***SE***	***B***	***SE***	***B***	***SE***	***B***	***SE***
Intercept	3.98 (<0.001)	0.02	3.99 (<0.001)	0.03	3.99 (<0.001)	0.03	2.66 (<0.001)	0.03	2.63 (<0.001)	0.03	2.64 (<0.001)	0.03
DJ	0.07 (0.011)	0.03	0.07 (0.013)	0.03	0.07 (0.028)	0.03	−0.18 (<0.001)	0.03	−0.17 (<0.001)	0.04	−0.15 (<0.001)	0.04
PJ	0.23 (<0.001)	0.03	0.23 (<0.001)	0.03	0.23 (<0.001)	0.03	−0.15 (<0.001)	0.04	−0.16 (<0.001)	0.04	−0.15 (<0.001)	0.04
AUT	0.11 (<0.001)	0.03	0.11 (<0.001)	0.03	0.10 (0.002)	0.03	−0.10 (0.006)	0.04	−0.10 (0.008)	0.04	−0.07 (0.077)	0.04
DJ × PJ			−0.03 (0.320)	0.03	−0.04 (0.240)	0.03			0.07 (0.043)	0.04	0.09 (0.013)	0.04
PJ × AUT			<0.01 (0.978)	0.04	<0.01 (0.956)	0.04			0.04 (0.423)	0.04	0.04 (0.382)	0.04
DJ × AUT			−0.05 (0.142)	0.04	−0.04 (0.307)	0.04			0.03 (0.460)	0.05	−0.01 (0.835)	0.05
DJ × PJ × AUT					0.04 (0.252)	0.04					−0.12 (0.005)	0.04
*R^2^*	0.09 (<0.001)		0.09 (<0.001)		0.09 (<0.001)		0.07 (<0.001)		0.07 (<0.001)		0.08 (<0.001)	
*R^2^ change*			<0.01 (0.218)		<0.01 (0.252)				<0.01 (0.054)		0.01 (0.005)	

*N = 1117. All predictors were centered on the grand mean prior to analyses. Numbers in parentheses are corresponding p-values. DJ, distributive justice; PJ, procedural justice; AUT, job autonomy*.

**Figure 3:**
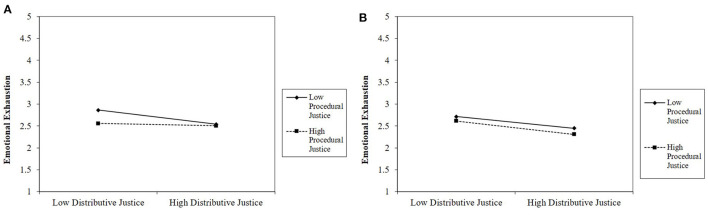
**(A)** The effect of distributive and procedural justice on emotional exhaustion when job autonomy is low (Study 2). **(B)** The effect of distributive and procedural justice on emotional exhaustion when job autonomy is high (Study 2).

Figures 3A,B depict the interaction for emotional exhaustion. In support of our hypothesis, simple slopes analyses showed that among employees with low job autonomy (1 *SD* below the mean), the interaction between distributive and procedural justice was significant (*B* = 0.19, *F*_(1,1109)_ = 12.00, *p* = 0.001). More specifically, among employees with low job autonomy, distributive justice was related to lower emotional exhaustion when procedural justice was low (1 *SD* below the mean; *B* = −0.29, *SE* = 0.05, *p* < 0.001) but not significantly related when procedural justice was high (1 *SD* above the mean *B* = 0.01, *SE* = 0.08, *p* = 0.912). From a different vantage point, among employees with low autonomy, the effect of procedural justice was significant when distributive justice was low (*B* = −0.35, *SE* = 0.07, *p* < 0.001) and not significant when distributive justice was high (*B* = −0.02, *SE* = 0.07, *p* = *0.8*16). Among employees with high job autonomy, the interaction between procedural and distributive justice was not significant (*B* < −0.01, *F*_(1,1109)_ = 0.01, *p* = 0.943).

##### Robustness Checks

Similar to what we did in Study 1, we first estimated models that did not include demographic variables as controls. We then estimated the same model as in the main text, including gender, age, and education level as predictors. The focal three-way interaction on emotional exhaustion (*B* = −0.11, *SE* = 0.04, *p* = 0.009) was still significant and of the same shape as in the main analyses section.

We conducted exploratory analyses to test if the role of job autonomy in moderating the Distributive Justice × Procedural Justice interaction is independent from possible covariation of job autonomy with power. Following previous research that used the number of subordinates as a measure of power (Sherman et al., 2012; Zhang et al., 2014), we used the number of direct subordinates as our proxy of power, where 0 implied that the participant had no supervisory role. The number of direct subordinates ranged from 0 to 99 in our sample (*M* = 1.83, *SD* = 7.30). We ran the full model presented in Table 6 (Model 3) and included the three-way interaction and all lower order effects between procedural justice, distributive justice, and number of direct subordinates. For emotional exhaustion the focal three-way interaction between distributive justice, procedural justice, and job autonomy remained significant and of the same shape as in the main analyses (*B* = −0.12, *SE* = 0.04, *p* = 0.006); the three-way interaction with the number of direct subordinates was not significant (*B* < −0.01, *SE* < 0.01, *p* = 0.500). For job satisfaction, the focal three-way interaction with autonomy remained nonsignificant (*B* = 0.03, *SE* = 0.04, *p* = 0.348); however, the three-way interaction with number of direct subordinates was significant (*B* = 0.01, *SE* < 0.01, *p* = 0.011). In sum, exploratory analyses including three-way interactions with the number of subordinates as a proxy of power did not affect our conclusions with regards to the focal three-way interaction effect between distributive justice, procedural justice, and autonomy on emotional exhaustion and job satisfaction.

## General Discussion

We showed across two field studies that the Distributive Justice × Procedural Justice interaction effect is moderated by job autonomy for two different but correlated indicators of employee's wellbeing. Specifically, among employees with low job autonomy, procedural justice moderated the relationship between distributive justice and job satisfaction (Study 1) and emotional exhaustion (Study 1 and 2) such that high procedural justice mitigated the effect of distributive justice. Among employees with high job autonomy, procedural justice did not moderate the relationship between distributive justice and these two wellbeing outcomes. We established this boundary role of job autonomy using validated measures of employee autonomy, procedural and distributive justice, job satisfaction, and emotional exhaustion. Below we discuss the implications and limitations of these findings.

### Theoretical Implications

The JDC model predicts that job control buffers against the effects of job demands (i.e., stressors) on employee wellbeing (Karasek, 1979). Similarly, previous work on organizational justice has argued that procedural justice buffers the negative effects of distributive injustice on employee wellbeing (Tepper, 2001; Vermunt and Steensma, 2005; Brockner, 2011). One way in which procedural justice acts as a buffer is that fair processes give employees (a feeling of) control (Thibaut and Walker, 1975; Judge and Colquitt, 2004). Following this work, we expected that the Distributive Justice × Procedural Justice interaction is more likely to emerge for employees that have lower job autonomy (i.e., lower control) than for those with higher job autonomy. We expected this as employees in low autonomous jobs should be more likely to rely on other resources that may provide them with some (feeling of) control, such as procedural justice (Van Prooijen, 2009). In line with this prediction, we show that the buffering effect of procedural justice depends on the level of job autonomy. We thus identified job autonomy as a novel and theoretically relevant boundary condition to the Distributive Justice × Procedural Justice interaction effect on wellbeing outcomes.

The present findings thus may contribute to explaining previous inconsistent findings regarding the Distributive Justice × Procedural Justice interaction effect for wellbeing outcomes. For instance, Tepper (2001) found that the interactive relationship predicted emotional exhaustion, depression, and anxiety. However, other studies did not find this interaction effect on related measures of wellbeing (McFarlin and Sweeney, 1992; Fischer et al., 2014). Our study suggests that one reason for the different findings between studies could be variations in the level of job autonomy between the studies involved.

For instance, Fischer et al. (2014), who did not find a significant interaction effect between distributive and procedural justice, excluded shop floor workers and employees doing manual labor. As these jobs often involve low levels of autonomy (Vidal, 2013), the resulting sample might primarily consist of employees with higher levels of autonomy. Tepper (2001) who, in contrast, did find a significant interaction effect in two studies, included participants from a wide range of jobs that includes low levels of autonomy, such as construction workers and clerical workers (Vidal, 2013). In addition, average levels of job autonomy have increased in the past few decades (Wegman et al., 2018). Differences between older papers with significant findings (Fields et al., 2000; Tepper, 2001) and more recent papers with non-significant findings (Fischer et al., 2014; cf. McFarlin and Sweeney, 1992) might therefore be due to different levels of job autonomy in the study samples.

Our findings correspond well with insights on the buffer effect proposed within the JDC literature (Häusser et al., 2010). A recent meta-analysis investigating the validity of the buffer effect found that this effect was more strongly related to wellbeing outcomes when demands refer to hindrances instead of challenges (Gonzalez-Mulé et al., 2021). Challenge demands are stressors that are energizing and provide an opportunity for achievement and learning (Cavanaugh et al., 2000; LePine et al., 2005); a few examples are time pressure and a high workload. Hindrance demands are stressors that offer undesirable constraints and thwart personal growth and goal attainment (Cavanaugh et al., 2000; Gonzalez-Mulé et al., 2021). Receiving unfair outcomes is an example of hindrance demands; it is unlikely to be beneficial and provide an opportunity to learn. As there is less research on the interaction between hindrance demands and control (Gonzalez-Mulé et al., 2021), our current study adds to this body of work and supports the view that hindrance demands and job control interact to predict employee wellbeing.

### Practical Implications

Overall, focusing on the interaction between distributive justice, procedural justice, and job autonomy presents practical implications that could be tested and implemented depending on the needs and resources of organizations. Viewed in this manner, the current study provides several new insights on *when* employees benefit most from a focus on justice.

Firstly, fair procedures matter a lot to employees when their outcomes involve little autonomy and are perceived by them as negative or unfair, such as is often the case following layoffs and negative promotion decisions. Research has shown that procedural justice is related to positive employee outcomes both for survivors and victims of job layoffs (Brockner et al., 1994). Some examples of procedural justice in these contexts are giving an advanced notice (Brockner et al., 1994), giving voice to employees, and ensuring consistency in decision-making (Brockner et al., 1995).

Secondly, organizations may well be advised to focus on protecting distributive justice when both procedural justice and job autonomy are low. Personnel screening and selection contexts (Cropanzano et al., 2007) provide a good example of such situations. Standardized and structured tests (i.e., personality and cognitive ability tests) are perceived to be procedurally less fair and to allow less autonomy than unstructured interviews (Nolan and Highhouse, 2014). Nonetheless, standardized tests are a better indicator of job performance, while the predictive validity of unstructured interviews is low and can even hurt personnel selection decisions (Kausel et al., 2016). To minimize the detrimental effects of lowered procedural justice and autonomy, organizations could increase distributive justice perceptions by emphasizing and communicating equity in selection decisions (Celani et al., 2008).

Thirdly, organizations could try to increase job autonomy when neither distributive nor procedural justice can be ensured. Organizations are not always successful in securing high justice. This may be due to, for instance, rapid and radical changes within organizations (Kickul et al., 2002) or to employees' characteristics that influence their justice perceptions (Lang et al., 2011). The current study suggests a strategy for protecting employees' wellbeing when justice is low: increasing job autonomy. For instance, job design research has demonstrated how organizations granting more autonomy to employees positively influence employees' job satisfaction and wellbeing (Humphrey et al., 2007). Small changes in job designs that increase job autonomy can have a substantial impact; for example, giving call-center workers more autonomy rather than rules on how to display facial expressions decreased emotional exhaustion (Goldberg and Grandey, 2007).

### Limitations and Suggestions for Future Research

Like all research, ours has limitations. One of these is that we cannot draw causal conclusions from our two studies because they relied on cross-sectional designs. However, it should be noted that several experimental studies (e.g., Van den Bos et al., 1997) have established the interactive effect of procedural and distributive justice on various outcomes. Thus, the literature makes us quite confident about the causal direction of these effects. This implies that our reliance on cross-sectional designs is less of a problem. Future research could test causal effects by manipulating the degree of autonomy, the fairness of outcomes, and the fairness of procedures, or use longitudinal designs to exclude some alternative causal explanations.

Furthermore, the single source nature of the studies could result in common method variance that undermines our theoretical explanation (Podsakoff et al., 2012). However, studies show that common method variance does not inflate interaction effects (Evans, 1985; Siemsen et al., 2010), which suggests that our results are not affected by method bias.

The current study shows that the effect of organizational justice on employee wellbeing may depend on a job characteristic—job autonomy. In so doing we answer calls for more research on the interplay between justice and organizational structure and job characteristics (Schminke et al., 2015). Organizational characteristics play a role in organizational justice perceptions and effects of organizational justice (for a review, see Schminke et al., 2015). For instance, jobs that are characterized by high degrees of formalization (i.e., work processes being uniformly structured) tend to be perceived as higher in distributive and procedural justice perceptions (Schminke et al., 2002) but lower in autonomy (Langfred and Rockmann, 2016). Future research should investigate effects of organizational characteristics on wellbeing-related outcomes taking into account job autonomy and justice.

### Concluding Remarks

Previous work has identified distributive justice and procedural justice as important factors that improve employee wellbeing. The current study set out to investigate when these two types of justice interact to predict wellbeing, identifying job autonomy as a moderator to the interactive relationship. The results of this study indicate that when employees have low job autonomy, fair procedures help them cope with the stressor of unfair outcomes, thus protecting their wellbeing. When employees have high job autonomy, fair procedures do not buffer unfair outcomes. Taken together, the findings in the current study provide new insights on *when* employees' wellbeing benefits most from a focus on justice, thus showing relevant theoretical and practical implications.

## Data Availability Statement

The raw data supporting the conclusions of this article will be made available by the authors, without undue reservation.

## Ethics Statement

Ethical review and approval was not required for the study on human participants in accordance with the local legislation and institutional requirements. The patients/participants provided their written informed consent to participate in this study.

## Author Contributions

LV conducted the analyses and wrote the manuscript together with MD. JR collected the data for Study 1 and Study 2. KB helped in writing the manuscript. All authors contributed to the article and approved the submitted version.

## Funding

This research was financed by the Open University of the Netherlands.

## Conflict of Interest

The authors declare that the research was conducted in the absence of any commercial or financial relationships that could be construed as a potential conflict of interest.

## Publisher's Note

All claims expressed in this article are solely those of the authors and do not necessarily represent those of their affiliated organizations, or those of the publisher, the editors and the reviewers. Any product that may be evaluated in this article, or claim that may be made by its manufacturer, is not guaranteed or endorsed by the publisher.

## References

[B1] Adams J. S. (1965). Inequity in social exchange, in Advances in Experimental Social Psychology, eds Berkowitz L. (New York, NY: Academic Press), p. 267–299.

[B2] Aguinis H. Gottfredson R. K. Joo H. (2013). Best-practice recommendations for defining, identifying, and handling outliers. Organization. Res. Methods 16, 270–301. 10.1177/1094428112470848

[B3] Aiken L. S. West S. G. (1991). Multiple Regression: Testing and Interpretinginteractions. Newbury Park, CA: Sage.

[B4] Bakker A. B. Demerouti E. Euwema M. C. (2005). Job resources buffer the impact of job demands on burnout. J. Occupation. Health Psychol. 10, 170–180. 10.1037/1076-8998.10.2.170 15826226

[B5] Bakotić D. Bulog I. (2021). Organizational justice and leadership behavior orientation aspredictors of employees job satisfaction: evidence from Croatia. Sustainability 13, 10569. 10.3390/su131910569

[B6] Belsley D. A. Kuh E. Welsch R. E. (1980). Regression Diagnostics: Identifying Influential Data and Sources Of Collinearity. New York, NY: John Wiley and Sons.

[B7] Bianchi E. C. Brockner J. Van den Bos K. Seifert M. Moon H. van Dijke M.. (2015). Trust in decision-making authorities dictates the form of theinteractive relationship between outcome fairness and procedural fairness. Personal. Soc. Psychol. Bull. 41, 19–34. 10.1177/0146167214556237 25387762

[B8] Blader S. L. Chen Y. R. (2012). Differentiating the effects of status and power: a justiceperspective. Journal of Personality and Social Psychology 102, 994–1014. 10.1037/a0026651 22229456

[B9] Brayfield A. H. Rothe H. F. (1951). An index of job satisfaction. J. Appl. Psychol. 35, 307-311.

[B10] Breaugh J. A. (1985). The measurement of work autonomy. Human Relat. 38, 551–570. 10.1037/h0055617

[B11] Brockner J. (2011). A Contemporary Look at Organizational Justice: Multiplying Insult Times Injury [Kindle version]. *Retrieved from* amazon.com

[B12] Brockner J. Konovsky M. Cooper-Schneider R. Folger R. Martin C. Bies R. J. (1994). Interactive effects of procedural justice and outcome negativity on victims andsurvivors of job loss. Acad. Manage. J. 37, 397–409. 10.2307/256835

[B13] Brockner J. Wiesenfeld B. M. (1996). An integrative framework for explaining reactionsto decisions: interactive effects of outcomes and procedures. Psychologic. Bull. 120, 189–208. 10.1037/0033-2909.120.2.189 8831296

[B14] Brockner J. Wiesenfeld B. M. (2005). How, when, and why does outcome favorabilityinteract with procedural fairness?, in Handbook of Organizational Justice, eds Greenberg J. Colquitt J. A. (Mahwah, NJ: Lawrence Erlbaum Associates), pp. 525–554.

[B15] Brockner J. Wiesenfeld B. M. Martin C. L. (1995). Decision frame, procedural justice,and survivor's reactions to job layoffs. Organization. Behav. Hum. Decis. Processes 63, 59–68. 10.1006/obhd.1995.1061

[B16] Brotheridge C. M. (2003). The role of fairness in mediating the effects of voice and justificationon stress and other outcomes in a climate of organizational change. Int. J. Stress Manage. 10, 253–268. 10.1037/1072-5245.10.3.253

[B17] Cavanaugh M. A. Boswell W. R. Roehling M. V. Boudreau J. W. (2000). An empiricalexamination of self-reported work stress among US managers. J. Appl. Psychol. 85, 65–74. 10.1037/0021-9010.85.1.65 10740957

[B18] Celani A. Deutsch-Salamon S. Singh P. (2008). In justice we trust: a model of the roleof trust in the organization in applicant reactions to the selection process. Hum. Resour. Manage. Rev. 18, 63–76. 10.1016/j.hrmr.2008.04.002

[B19] Centraal Bureau Statistiek. (2022). Statline. Available online at: https://opendata.cbs.nl/#/CBS/nl/ (accessed March 24, 2022).

[B20] Chen Y. R. Brockner J. Greenberg J. (2003). When is it “a pleasure to do business withyou?” The effects of relative status, outcome favorability, and procedural fairness. Organization. Behav. Hum. Decis. Process. 92, 1–21. 10.1016/S0749-5978(03)00062-1

[B21] Cohen J. E. (1988). Statistical Power Analysis for the Behavioral Sciences. Hillsdale, NJ: LawrenceErlbaum Associates, Inc

[B22] Cohen-Charash Y. Spector P. E. (2001). The role of justice in organizations: a meta-analysis. Organization. Behav. Hum. Decis. Processes 86, 278–321. 10.1006/obhd.2001.295830730164

[B23] Colquitt J. A. (2001). On the dimensionality of organizational justice: a construct validation of a measure. J. Appl. Psychol. 86, 386–400. 10.1037/0021-9010.86.3.386 11419799

[B24] Colquitt J. A. Conlon D. E. Wesson M. J. Porter C. O. L. H. Yee K. (2001). Justice atthe millennium. a meta-analytic review of 25 years of organizational justice research. J. Appl. Psychol. 86, 425–445. 10.1037/0021-9010.86.3.425 11419803

[B25] Colquitt J. A. Scott B. A. Rodell J. B. Long D. M. Zapata C. P. Conlon D. E.. (2013). Justice at the millennium, a decade later: a meta-analytic test of social exchange and affect-based perspectives. J. Appl. Psychol. 98, 199–236. 10.1037/a0031757 23458336

[B26] Cropanzano R. Bowen D. E. Gilliland S. W. (2007). The management of organizationaljustice. Acad. Manage. Perspect. 21, 34–48. 10.5465/amp.2007.27895338

[B27] Danna K. Griffin R. W. (1999). Health and wellbeing in the workplace: a review andsynthesis of the literature. J. Manage. 25, 357–384. 10.1177/014920639902500305

[B28] De Jonge J. Schaufeli W. B. (1998). Job characteristics and employee well-being: a testof Warr's Vitamin Model in health care workers using structural equation modelling. J. Organization. Behav. Int. J. Industr. Occupation. Organization. Psychol. Behav. 19, 387–407.

[B29] Evans M. G. (1985). A Monte Carlo study of the effects of correlated method variance inmoderated multiple regression analysis. Organization. Beha. Hum. Decis. Processes 36, 305–323. 10.1016/0749-5978(85)90002-0

[B30] Faul F. Erdfelder E. Buchner A. Lang A.-G. (2009). Statistical power analyses using G^*^Power3.1: Tests for correlation and regression analyses. Behav. Res. Methods 41, 1149–1160. 10.3758/BRM.41.4.1149 19897823

[B31] Fields D. Pang M. Chiu C. (2000). Distributive and procedural justice as predictors ofemployee outcomes in Hong Kong. J. Organization. Behav. 21, 547–562.

[B32] Fischer R. Abubakar A. Nyaboke Arasa J. (2014). Organizational justice and mentalhealth: a multi-level test of justice interactions. Int. J. Psychol. 49, 108–114. 10.1002/ijop.12005 24811881

[B33] Goldberg L. S. Grandey A. A. (2007). Display rules versus display autonomy: emotionregulation, emotional exhaustion, and task performance in a call center simulation. J. Occupation. Health Psychol. 12, 301–318. 10.1037/1076-8998.12.3.301 17638495

[B34] Gonzalez-Mulé E. Kim M. Ryu J. W. (2021). A meta-analytic test of multiplicative andadditive models of job demands, resources, and stress. J. Appl. Psychol. 106, 1391–1411. 10.1037/apl0000840 32955269

[B35] Goudswaard A. Dhondt S. Kraan K. (1998). Flexibilisering en arbeid in deinformatiemaatschappij; werknemersvragenlijst, bestemd voor werknemers enbedrijven die deelnemen aan het SZW-Werkgeverspanel 1998. [Flexibility and workin the information society; employers questionnaire, designed for employees and firmsthat participate at the SZW-Employers panel 1998]. TNO Arbeid.

[B36] Greenberg J. (2006). Losing sleep over organizational injustice: attenuating insomniac reactions tounderpayment inequity with supervisory training in interactional justice. J. Appl. Psychol. 91, 58–69. 10.1037/0021-9010.91.1.58 16435938

[B37] Guest D. E. Isaksson K. De Witte H. (2010). Employment Contracts,Psychological Contracts, and Employee WellBeing: An International Study. New York, NY: Oxford University Press.

[B38] Haar J. M. Spell C. S. (2009). How does distributive justice affect work attitudes? themoderating effects of autonomy. Int. J. Human Resour. Manage. 20, 1827–1842. 10.1080/09585190903087248

[B39] Häusser J. A. Mojzisch A. Niesel M. Schulz-Hardt S. (2010). Ten years on: a review of recentresearch on the job demand–control (-support) model and psychological wellbeing. Work Stress 24, 1–35. 10.1080/02678371003683747

[B40] Hayes A. F. (2018). Introduction to Mediation, Moderation, and Conditional Process Analysis: A Regression-Based Approach (2nd ed.). New York, NY: Guilford Publications.

[B41] Horn J. E. van Schaufeli W. B. (1998). Maslach Burnout Inventory: The Dutch EducatorsSurvey (MBI-NLES) Psychometric Evaluations. Manual [unpublished manuscript]. Utrecht, Utrecht University: Department of Social and Organizational Psychology.

[B42] Humphrey S. E. Nahrgang J. D. Morgeson F. P. (2007). Integrating motivational, social,and contextual work design features: a meta-analytic summary and theoreticalextension of the work design literature. J. Appl. Psychol. 92, 1332–1356. 10.1037/0021-9010.92.5.1332 17845089

[B43] Judge T. A. Colquitt J. A. (2004). Organizational justice and stress: the mediating role ofwork-family conflict. J. Appl. Psychol. 89, 395–404. 10.1037/0021-9010.89.3.395 15161400

[B44] Karasek R. (1979). Job demands, job decision latitude, and mental strain: Implicationsfor job redesign. Administrat. Sci. Q. 24, 285–308. 10.2307/2392498

[B45] Karasek R. (1985). Job Content Questionnaire and User's Guide. Lowell: University of Massachusetts, Department of Work Environment.

[B46] Kausel E. E. Culbertson S. S. Madrid H. P. (2016). Overconfidence in personnelselection: When and why unstructured interview information can hurt hiring decisions. Organization. Behav. Human Decis. Process. 137, 27–44. 10.1016/j.obhdp.2016.07.005

[B47] Kickul J. Lester S. W. Finkl J. (2002). Promise breaking during radical organizationalchange: do justice interventions make a difference? J. Organization. Behav. 23, 469–488. 10.1002/job.151

[B48] Lammers J. Stoker J. I. Rink F. Galinsky A. D. (2016). To have control over or to befree from others*?* the desire for power reflects a need for autonomy. Personal. Soc. Psychol. Bull. 42, 498–512. 10.1177/0146167216634064 26984014

[B49] Lang J. Bliese P. D. Lang J. W. Adler A. B. (2011). Work gets unfair for thedepressed: cross-lagged relations between organizational justice perceptions and depressivesymptoms. J. Appl. Psychol. 96, 602–618. 10.1037/a0022463 21299270

[B50] Langfred C. W. Rockmann K. W. (2016). The push and pull of autonomy: The tension betweenindividual autonomy and organizational control in knowledge work. Group Organization Manage. 41, 629–657. 10.1177/1059601116668971

[B51] LePine J. A. Podsakoff N. P. LePine M. A. (2005). A meta-analytic test of the challengestressor–hindrance stressor framework: an explanation for inconsistent relationships among stressors and performance. Acad. Manage. J. 48, 764–775. 10.5465/amj.2005.18803921

[B52] Maslach C. Jackson S. E. (1981). The measurement of experienced burnout. J. Organ. Behav. 2, 99–113. 10.1002/job.4030020205

[B53] Maslach C. Jackson S. E. (1986). Maslach Burnout Inventory (2 ed.). Palo Alto, CA: ConsultingPsychologists Press.

[B54] McFarlin D. B. Sweeney P. D. (1992). Distributive and procedural justice as predictors ofsatisfaction with personal and organizational outcomes. Acad. Manage. J. 35, 626–637. 10.5465/256489

[B55] Nolan K. P. Highhouse S. (2014). Need for autonomy and resistance to standardizedemployee selection practices. Hum. Performan. 27, 328–346. 10.1080/08959285.2014.92969128476160

[B56] Podsakoff P. M. MacKenzie S. B. Podsakoff N. P. (2012). Sources of method bias insocial science research and recommendations on how to control it. Ann. Rev. Psychol. 63, 539–569. 10.1146/annurev-psych-120710-100452 21838546

[B57] Robbins J. M. Ford M. T. Tetrick L. E. (2012). Perceived unfairness and employeehealth: a meta-analytic integration. J. Appl. Psychol. 97, 235–272. 10.1037/a0025408 21928872

[B58] Rousseau V. Salek S. Aub,é C. Morin E. M. (2009). Distributive justice, proceduraljustice, and psychological distress: the moderating effect of coworker support andwork autonomy. J. Occupation. Health Psychol. 14, 305–317. 10.1037/a0015747 19586224

[B59] Schaufeli W. B. Daamen J. Van Mierlo H. (1994). Burnout among Dutch teachers: AnMBI-validity study. Educ. Psychologic. Measure. 54, 803–812. 10.1177/0013164494054003027

[B60] Schminke M. Cropanzano R. Rupp D. E. (2002). Organization structure and fairness perceptions: the moderating effects of organizational level. Organ. Behav. Human Decis. Processes. 89, 881–905. 10.1016/S0749-5978(02)00034-1

[B61] Schminke M. Johnson M. A. Rice D. (2015). Justice and organizational structure: A review, in The Oxford Handbook of Justice in the Workplace, eds. Cropanzano R. S. Ambrose M. L. (New York, NY: Oxford University Press), p. 541–560.

[B62] Sheeraz M. I. Ahmad U. N. U. Ishaq M. I. Sarfraz M. Nor K. M. (2021). The research onorganizational justice in scopus indexed journals: a bibliometric analysis of seven decades. Front. Psychol. 12, 6485. 10.3389/fpsyg.2021.647845 34177702 PMC8222511

[B63] Sherman G. D. Lee J. J. Cuddy A. J. Renshon J. Oveis C. Gross J. J. Lerner J. S. (2012). Leadership is associated with lower levels of stress. Proceed. Nat. Acad. Sci. 109, 17903–17907. 10.1073/pnas.1207042109 23012416 PMC3497788

[B64] Siemsen E. Roth A. Oliveira P. (2010). Common method bias in regression models withlinear, quadratic, and interaction effects. Organization. Res. Methods 13, 456–476. 10.1177/1094428109351241

[B65] Spector P. E. Brannick M. T. (2011). Methodological urban legends: the misuse ofstatistical control variables. Organization. Res. Methods 14, 287–305. 10.1177/1094428110369842

[B66] Tepper B. J. (2001). Health consequences of organizational injustice: tests of main andinteractive effects. Organization. Behav. Hum. Decis. Processes, 86, 197–215. 10.1006/obhd.2001.2951

[B67] Theorell T. Karasek R. A. (1996). Current issues relating to psychosocial job strain andcardiovascular disease research. J. Occupation. Health Psychol. 1, 9–26. 10.1037/1076-8998.1.1.9 9547038

[B68] Thibaut J. Walker L. (1975). Procedural Justice: A Psychological Analysis. Hillsdale, NJ: Erlbaum.

[B69] Van den Bos K. Vermunt R. Wilke H. A. (1997). Procedural and distributive justice:What is fair depends more on what comes first than on what comes next. J. Personal. Soc. Psychol. 72, 95–104. 10.1037/0022-3514.72.1.95

[B70] Van Dijke M. Gobena L. Verboon P. (2019). Make me want to pay. a three-wayinteraction between procedural justice, distributive justice, and power on voluntary taxcompliance. Front. Psychol. 10, 1632. 10.3389/fpsyg.2019.01632 31354602 PMC6639977

[B71] Van Prooijen J. W. (2009). Procedural justice as autonomy regulation. J. Personal. Soc. Psychol. 96, 1166–1180. 10.1037/a0014153 19469594

[B72] Vermunt R. Steensma H. (2003). Physiological relaxation: stress reduction through fairtreatment. Soc. Justice Res. 16, 135–149. 10.1023/A:1024200120646

[B73] Vermunt R. Steensma H. (2005). How can justice be used to manage stress inorganizations, in Handbook of Organizational Justice, eds Greenberg J. Colquitt J. A. (Hillsdale, NJ: Lawrence Erlbaum Associates), pp. 383–410.

[B74] Vidal M. (2013). Low-autonomy work and bad jobs in postfordist capitalism. Humanrelations 66, 587–612. 10.1177/0018726712471406

[B75] Wanous J. P. Reichers A. E. Hudy M. J. (1997). Overall job satisfaction: how good aresingle-item measures? J. Appl. Psychol. 82, 247–252. 9109282 10.1037/0021-9010.82.2.247

[B76] Wegman L. A. Hoffman B. J. Carter N. T. Twenge J. M. Guenole N. (2018). Placing jobcharacteristics in context: cross-temporal meta-analysis of changes in job characteristicssince 1975. J. Manage. 44, 352–386. 10.1177/0149206316654545

[B77] Weiss H. M. Suckow K. Cropanzano R. (1999). Effects of justice conditions on discreteemotions. J. Appl. Psychol. 84, 786–794. 10.1037/0021-9010.84.5.78626651622

[B78] Zhang A. Y. Song L. J. Tsui A. S. Fu P. P. (2014). Employee responses to employment-relationship practices: the role of psychological empowerment and traditionality. J. Organization. Behav. 35, 809–830. 10.1002/job.1929

